# New approach to intermittent and mild asthma therapy: evolution or revolution in the GINA guidelines?

**DOI:** 10.1186/s13601-020-00316-z

**Published:** 2020-06-03

**Authors:** Izabela Kuprys-Lipinska, Marta Kolacinska-Flont, Piotr Kuna

**Affiliations:** Department of Internal Medicine, Asthma and Allergy, Norbert Barlicki University Hospital in Lodz, Medical University of Lodz, 22 Kopcinskiego Str., 90-153 Lodz, Poland

**Keywords:** GINA guidelines, Symptom-driven therapy, Short-acting β2 agonist, Inhaled corticosteroids

## Abstract

New recommendations from the Global Initiative for Asthma (GINA) were released in a pocket guide form on April 12, 2019. These recommendations provide very important changes to the management of asthma, especially regarding the treatment of intermittent and mild asthma. Due to safety concerns, GINA experts no longer recommend treatment with a short-acting β2 agonist alone. Henceforth, all adults and adolescents (but not yet children) with mild asthma should receive either symptom-driven or daily low-dose ICS. The main goal of this new approach is to reduce the risk of serious asthma exacerbations and asthma-related deaths in the population of patients with mild asthma. Herein, the authors present the epidemiological and clinical data regarding the risks of excessive SABA use and the benefits of regular treatment with inhaled corticosteroids. The authors deliver a critical review on the evolution of the changes in the GINA experts’ standpoint and provide evidence-based background for the new approach to asthma treatment. Moreover, the authors identify gaps and unmet needs still present in the current asthma management recommendations and discuss them thoroughly.

## Background

On April 12, 2019, new recommendations from the Global Initiative for Asthma (GINA) were released [1]. Initially, the report was available only in a shortened form (a “pocket guide”), yet it heralded a long-awaited breakthrough in asthma management, especially regarding the approach to intermittent and mild asthma treatment.

According to GINA experts, changes proposed in the 2019 report are the most fundamental change to asthma therapy in the last 30 years, which is approximately since the first guidelines were developed.

In brief, currently experts recommend introducing anti-inflammatory treatment at the very initiation of asthma therapy, i.e., all adults and adolescents with mild asthma should receive low-dose inhaled corticosteroids (ICS) either for symptom-driven use or as a regular daily medication to reduce the risk of serious exacerbations. The withdrawal of the recommendation for on-demand short-acting β2 agonists (SABA) as monotherapy as the first step of asthma treatment and the introduction/addition of symptom-driven ICS treatment on the first/second step of asthma therapy is the major paradigm shift from the previous GINA report.

The main goal of these changes is to reduce the risk of serious asthma exacerbations and asthma-related deaths in the population of patients with mild asthma. It has been widely known that SABA medications, while they provide quick relief from asthma symptoms, provide no anti-inflammatory effects and therefore do not treat the underlying cause of airway constriction. Consequently, when used alone, SABA treatments do not prevent severe exacerbations. Moreover, their regular or frequent use even increases the risk of near-fatal and fatal asthma attacks.

Herein, the authors present the epidemiological and clinical data regarding the risks of excessive SABA use and the benefits of adding of inhaled corticosteroids to the asthma therapy. The authors deliver a critical review on the evolution of the changes in the GINA experts’ standpoint since 1995 till 2019 regarding SABA usage and show the development of new concept in management of intermittent and mild asthma. Authors provide evidence-based background for use of budesonide-formoterol (BUD-FORM) as a rescue medication and present the pros and cons of such regimen. Moreover, the authors identify gaps and unmet needs still present in the current asthma management recommendations and discuss them thoroughly.

## Epidemiological data and clinical studies on excessive use of SABA

**Fig. 1:**
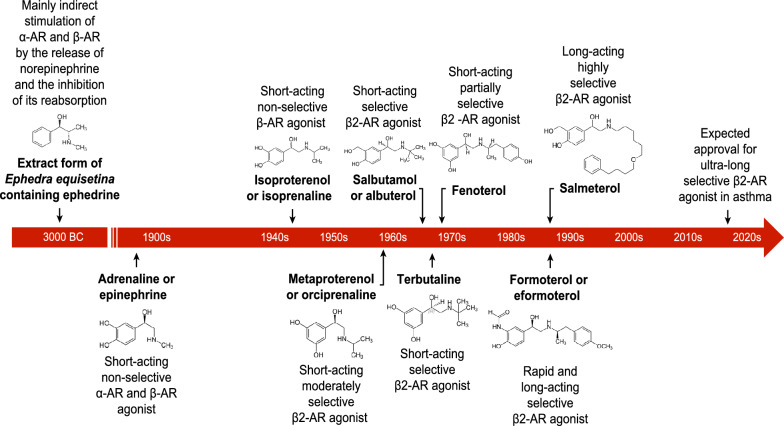
Timeline for the introduction of adrenergic receptor agonists in asthma treatment [2–16]. *AR* adrenoceptor

**Fig. 2:**
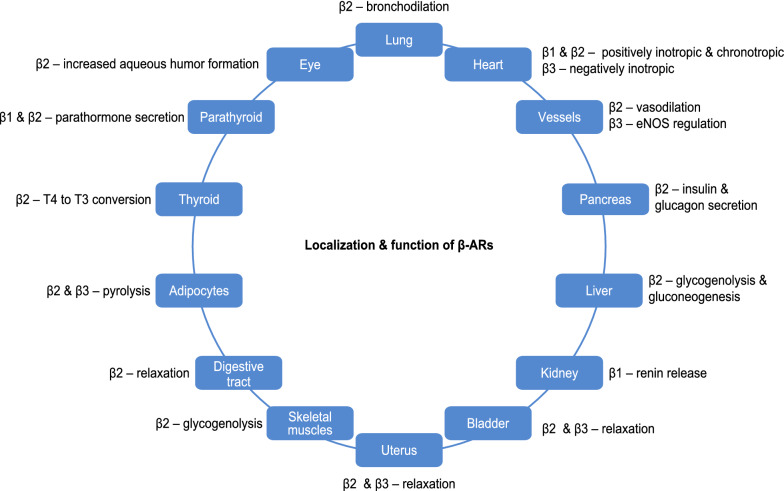
Localization and function of β-adrenoceptors [17, 18]. *AR* adrenoceptors

**Fig. 3:**
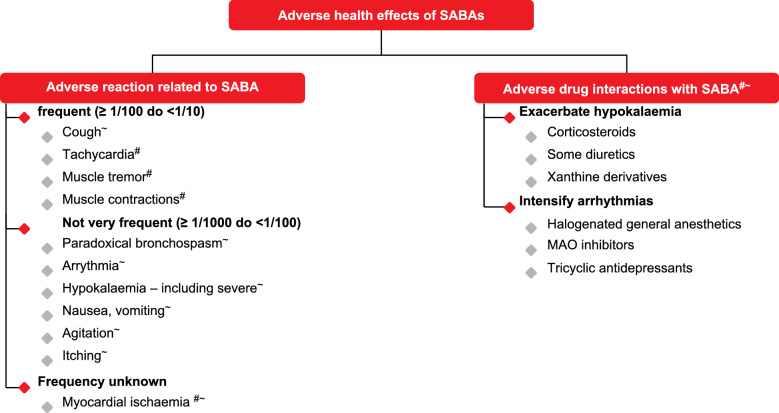
Adverse effects and harmful drug interaction in patients using SABA. #salbutamol [21], ~ fenoterol [22]

The use of adrenoceptor (AR) agonists dates back to 3000 BC when Chinese medicine practitioners used *ma huang* (*Ephedra equisetina*) extracts containing ephedrine in the treatment of respiratory symptoms [2]. At the beginning of twentieth century, the nonselective α-AR and ß-AR agonist epinephrine was introduced into clinical practice and administered by the subcutaneous route for the treatment of acute asthma [3, 4]. Although highly efficacious, epinephrine caused serious adverse event due to its effect on α and ß -ARs in the cardiovascular system. Isoprenaline [5] and metaproterenol [6] were next AR agonists interacting only with ß-AR but non-selective to their subtypes (Fig. 1) [2–16]. Their administration was complicated by cardiac adverse events as they did not discriminate between ß1- and ß2-ARs (Fig. 2) [17, 18]). The development of selective ß2-AR agonists salbutamol [7, 8], terbutaline [9, 10] and fenoterol [11] started the modern era of short acting ß2 agonists (SABA) (Fig. 1). These drugs were used by inhalation route thanks to the construction of the first personal inhalers in 1940s–1950s [19, 20]. High efficiency in relieving acute bronchospasm resulted in the popularity of these drugs and their market increased rapidly all over the world, but quite early, there were doubts about their safety due to numerous side effects, especially serious affecting the circulatory and respiratory systems (Fig. 3 [21, 22]).

The notorious reputation of SABA therapies dates back to the 1970s, when soon after their introduction to the market, an increase in asthma-related mortality was observed. A series of papers published during that time alarmed the public to the epidemic of sudden deaths due to asthma in England, Wales, Scotland, Ireland, Australia and New Zealand and linked it to an increase in the consumption of isoprenaline (a nonselective β mimetic) [23] and then fenoterol (classified as a selective β2 mimetic but with some affinity for β1 receptors) [24, 25]. The mechanism behind this correlation was not known, but in 1989 in New Zealand, fenoterol was withdrawn from the market, which resulted in a reduction in the country’s asthma-related mortality [26].

In the early 1990s, Spitzer et al. [27] reviewed the Saskatchewan Canadian province database and found an association between the regular use of SABA and morbidity and mortality in asthmatics. The authors proved that SABA use was associated with an increased risk of death from asthma (odds ratio, 2.6 per canister per month, 95% CI 1.7–3.9). They concluded that, regardless of whether or not beta-agonists are directly responsible for these serious side effects or are simply a marker of more severe asthma, the excessive use of SABA by patients should alarm providers to the urgent need of patient status reassessment.

Two years later, the same team [28] published results of an epidemiological analysis showing an association between the use of fenoterol, salbutamol and oral corticosteroids in the previous year and sudden asthma deaths as well as between the number of hospitalizations due to asthma in the previous 2 years and fatal cases. They also calculated that the risk of death increased dramatically after exceeding the usage of 1.4 SABA canisters per month.

In 1998, this team issued another publication [29] analyzing the reasons for hospital admissions due to asthma. The investigators observed that the inclusion of regular therapy with ICS in the year in which asthma was diagnosed reduced the risk of hospital admission by 40% compared with theophylline therapy. In 2000, Suissa et al. [30] reported that the use of low doses of ICS reduced the risk of sudden death from asthma. They calculated that the rate of death from asthma decreased by 21% with each additional canister of ICS used in the previous year. However, the mortality rate of patients who discontinued ICS therapy increased quickly within the first 3 months of treatment discontinuation.

In the same year, two more interesting studies were published. Hancox et al. [31] showed that the regular use of terbutaline led to the development of tolerance to the bronchodilating effect of the drug, and the discontinuation of such treatment caused rebound bronchoconstriction. In the second study, Aldridge et al. [32] showed that regular, high-dose terbutaline monotherapy increased eosinophil infiltration in the bronchi compared to the placebo group; however, using the same doses of terbutaline concurrently with budesonide reduced the initial eosinophilia. Both of these studies provided insight into the pathomechanism of adverse reactions associated with SABA monotherapy and warned against the use of SABA monotherapy, but the recommendations from GINA experts to use this treatment regimen in patients with sporadic and mild asthma remained unchanged.

Again, great concerns over the use of β2 mimetics surfaced in 2006 when the results of the SMART study (Salmeterol Multicenter Asthma Research Trial) were published [33]. In this study, salmeterol, a long-acting β2-agonist (LABA), was included as an add-therapy to previous asthma treatments. The study was conducted on a very large population of asthmatic patients (26,000). Statistical analysis showed a small but statistically significant increase in mortality and life-threatening events associated with asthma in the group of patients using salmeterol. This research sparked a new discussion on the association of these events with the use of LABA, and connections to the negative experiences with SABA were drawn. It was concluded that the cause of these deaths may have been inadequate anti-inflammatory treatment in these patients, because 9 out of 13 reported deaths occurred in patients who were not using ICS at the time of salmeterol inclusion. This finding reinforced the recommendations of GINA experts to use LABA only with ICS and to prefer a fixed combination of ICS-LABA, but it did not change their position on SABA monotherapy at the first stage of asthma treatment. Thus, a question arises: Why weren’t these SABA recommendations changed earlier?

Intermittent and mild asthma was considered to be primarily a disease of bronchoconstriction, although in 1988, Wardlaw et al. [34] had already shown that both symptomatic and asymptomatic mild asthmatics had airway inflammation. Using bronchoalveolar lavage (BAL), they found a significantly increased percentage of mast cells in all asthmatics and a significant elevation in eosinophil count and in the concentration of major basic protein (MBP) in the group of symptomatic patients. They also showed an inverse correlation between PC20 and the percentage of mast cells (p < 0.01), eosinophils (p < 0.05), epithelial cells (p < 0.05), and the amount of MBP in BAL (p < 0.01). In 1990, Foresi et al. [35] confirmed the occurrence of marked airway inflammation in asymptomatic asthmatic patients. They performed bronchoscopy, bronchial biopsies and BAL in young lifetime nonsmoking subjects with a history of asthma who had diurnal PEF variability lower than 20% and were free from acute respiratory infections or spontaneous asthma attacks within the previous month. These patients controlled their asthma symptoms with only SABA as needed or on a daily basis and held off using their medication for a 24-h period before the bronchoscopy. The investigators found greater cell infiltration of the epithelium and submucosa in the asthmatic subjects compared with healthy subjects. Additionally, eosinophils and intraepithelial mast cells were higher in the asthmatic group. A thickened basement membrane was associated with more marked cell infiltration in the submucosa. The cells in BAL broadly reflected cell infiltration of the submucosa, and the degree of bronchial responsiveness was correlated with ciliated cells in BAL and with intraepithelial cells in bronchial biopsies.

## The previous GINA recommendation for intermittent and mild asthma management

**Fig. 4:**
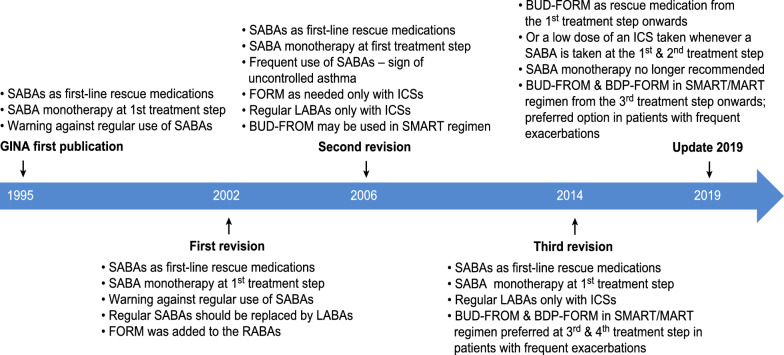
Timeline for SABA position changes in asthma management according to the GINA guidelines 1995–2019 [36, 37]. *ICS* inhaled corticosteroids, *LABA* long acting beta2 agonist, *RABA* rapid acting beta2 agonist, *SABA* short acting beta2 agonist, *SMART* single inhaler maintenance and rescue therapy, *MART* maintenance and rescue therapy, *BUD* budesonide, *BDP* beclomethasone, *FORM* formoterol

Despite obvious evidence of airway inflammation in patients with mild asthma, SABA held a strong position in the GINA recommendations from 1995 until 2019 (Fig. 4) [36, 37]. The only changes over the years concerned the recommended maximum number of SABA inhalations during the first step of treatment, which varied from 1 inhalation per week in 1995 to 2 inhalations per week in 2006 and fewer than 2 inhalation per month in 2014. In 2002, formoterol, a long-acting β2-agonist with a rapid onset of action was recommended as a rescue medication but only in patients receiving ICS. The question then arises: Why was SABA not also considered as a rescue medication for use only in patients using ICS? In 2014, a fixed combination of ICS with formoterol (budesonide and beclomethasone) was recommended as a rescue medication from the third step of treatment in patients receiving such medications as a maintenance therapy but not during the first or second steps, where a SABA was the only preferred rescue medication [36].

In the late 1990s, two big international epidemiological studies were carried out (ARIE [38] and Asthma in America [39]), which indicated the need for changes in asthma management. They showed that, despite the progress in pharmacological therapy and the updated recommendations for asthma management, most patients were symptomatic, with up to 50% feeling significantly impaired in their activities of daily living due to asthma, approximately 11–23% making emergency department visits in the previous year, and 7–9% having severe exacerbations that required hospitalization. Moreover, patients relied too heavily on SABAs and did not use ICS as they should have (while 63% of patients used a SABA, only 23% used an ICS).

Since the publication of the first guidelines in 1995, many important changes have been introduced to improve asthma outcomes. In 2006, the severity classification was changed to assess control when making therapeutic decisions, and single inhaler maintenance and reliever therapy (SMART) was recommended. The management of intermittent and mild asthma was unchanged, however, and remained consistent from when SABAs were introduced for use approximately 50 years ago.

Moreover, later cross-sectional studies showed that, despite the very important changes in GINA recommendations, asthma control was still suboptimal in approximately 50% (45–56, 5%) of patients. Severe exacerbations were still common and had occurred in 44% of patients within the previous year; 24% of those patients had needed an emergency department visit, and 12% had been hospitalized [40]. Uncontrolled asthma was the reason for a poor asthma-related quality of life (QoL), a high risk of exacerbations and significant consumption of healthcare resources [41].

It was a sign that there was a need for further changes in recommendations.

## New concept in management of intermittent and mild asthma

The epidemiological data proved that mild, intermittent, and persistent asthma are the most frequent forms of the disease and concern more than 50% of patients (ARIA), which explains their great impact on the overall asthma burden.

### Risk of severe exacerbations in mild asthma

In 2007, Dusser et al. [42] published a review showing that patients suffering from mild asthma quite often experience severe exacerbations at a frequency ranging from 0.12 to 0.77 per patient per year. Severe exacerbations in mild asthma represent as many as 30–40% of asthma exacerbations requiring emergency consultation. Even patients with mild symptoms meeting the criteria of asthma controlled by GINA are at risk of severe exacerbations (experiencing symptoms varying from 0 to 1–2 times per week). They constituted more than 70% of patient consults for acute asthma and patients with acute near fatal asthma in emergency departments.

### Risk of asthma exacerbation in patients overusing SABA or using them in monotherapy

Later, Stanford et al. [43] determined the magnitude of the risk of asthma exacerbation depending on SABA demand. Based on two large databases, researchers calculated that the consumption of 3 or more SABA canisters within the previous year increased the risk of asthma exacerbation. For adults, such a marker may be the use of 2 or more SABA canisters in a shorter 3–6 month period. The use of an additional SABA canister resulted in a corresponding increase in the risk of asthma exacerbation by 8% to 14% and 14% to 18% in children and adults, respectively.

In a survey conducted by Price et al. [44] in eight Asian countries, only 14.5% of respondents taking exclusively rescue medications had controlled asthma, and two-thirds had experienced severe exacerbations in the previous year. Unfortunately, in this study, similar proportions of adverse events were also found in the group taking controller medications, which may be because respondents confess that they fear the regular use of asthma drugs and then only take them occasionally; additionally, most of these patients could not correctly identify drugs for asthma prescribed by doctors.

There is one more interesting study from 1997 performed by Donahue et al. [45]. The investigators showed that SABA use was associated with an increased risk of hospitalization. The overall relative risk (RR) of hospitalization among those who received inhaled steroids was low and was 0.5 (95% confidence interval [CI], 0.4–0.6) after adjusting for SABA dispensing. The steroid-associated protection was most marked among individuals who received the largest amount of SABA, while the group of patients using eight or more SABA inhalations per day and ICS as maintenance therapy experienced a nearly 75% decrease in the risk of exacerbations compared to patients using only SABA. A fixed combination of SABA/rapid acting beta2 agonists (RABA) and ICS as a rescue medication therefore may protect against severe exacerbations.

### History of the BUD-FORM as needed concept

In 2008, in their review on the position of BUD-FORM, Kuna and Kupryś-Lipińska [46] proposed an extension of SMART and the inclusion of BUD-FORM as the medication to be used on demand from the first step of therapy according to the GINA guidelines. BUD-FORM serving as a rescue medication during the first and second steps of treatment could be a smooth transition to SMART in the further steps. The idea of using BUD-FORM on demand as the preferred therapy for the first and second steps of treatment was based on the clinical experience of the authors as well as the previous clinical studies and the knowledge of patient preferences and behavior, which were presented in the review.

Studies using formoterol or BUD-FORM as a rescue drug provided the most important support for this concept.

### Pharmacological basis of the BUD-FORM as needed concept

In 1996, Schreurs et al. [47] demonstrated the dose-dependent effect of formoterol (6, 12 and 24 µg#) in the maintenance therapy of patients with moderate asthma. Compared to placebo, 6 μg formoterol b.i.d. was found to be the lowest effective dose for improving the morning (p = 0.008) and evening (p = 0.0041) peak expiratory flow (PEF). Increasing the dose from 6 to 24 μg b.i.d. provided an additional effect of 18 L min^−1^ (p = 0.035) in the evening PEF. In 1998, Malolepszy et al. [48] demonstrated the effectiveness and safety of formoterol at high doses in the treatment of acute severe airway obstruction. Adult patients with asthma and COPD who had been admitted to intensive care units with acute severe bronchoconstriction (FEV1 = 20–50% of the predicted value) were randomized to receive 20 inhalations of either formoterol (4.5 μg^) or terbutaline (0.5 mg) within the first 3 h of therapy, but all received 40 mg of methylprednisolone i.v. in the 90th min of therapy. High doses of formoterol were equally as effective as terbutaline in improving lung function. Additionally, formoterol therapy resulted in significantly lower pulse rates than terbutaline therapy, which confirmed the safety profile of formoterol administered in high doses. Five years later, a similar study was performed comparing formoterol with salbutamol in an emergency setting [49]. In this study, using 54 µg^ of formoterol compared to 3600 µg of salbutamol resulted in greater acute lung function improvement over the 4-h assessment period.

In 2001, Palmqvist et al. [50] showed the rapid onset of bronchodilation (within the first 3 min) after inhalation of BUD-FORM (160/4.5 µg^ one or two doses). This effect was not observed after FLUT-SALM administration compared to placebo.

Another group of investigators [51] confirmed the immediate bronchodilation following BUD-FORM in patients with methacholine-induced moderate-to-severe bronchoconstriction and demonstrated rapid improvement of dyspnea (1 min after inhalation) as well as a shorter recovery time to 85% baseline lung function (approximately 3 min) than FLUT-SALM (approximately 9 min) or the placebo (30 min).

### Clinical evidence from first short term studies supporting the BUD-FORM as needed concept

In 2006, two very interesting studies on the use of BUD-FORM as a rescue medication were published. In Bateman et al. [52] study, budesonide-formoterol (with a total dose of 1280/36 µg^) and formoterol (with a total dose of 36 µg^) provided similarly rapid relief of acute bronchoconstriction in patients with asthma who were previously refractory to SABA treatment. The second study was SOMA, performed by Haahtela et al. [53] The investigators compared the as-needed use of RABA (formoterol 4.5 µg^) with the as-needed use of a RABA and corticosteroid fixed combination (BUD-FORM 160/4.5 µg^) as the only medication in asthma patients with intermittent symptoms. The study population consisted of patients who had previously only used RABA as needed with FeNO > 20 ppb. Baseline FeNO was 60 ppb and 59 ppb in the BUD-FORM and formoterol groups, respectively. During the 24 weeks of the study, FeNO was significantly reduced in patients receiving a combination of drugs from the fourth week of therapy until the end. The number of days of rescue medication use was significantly lower in the BUD-FORM group compared to the FORM group (21 days compared to 74 days). The authors concluded that the as-needed use of ICS-RABA may be more beneficial than using RABA alone in patients with intermittent asthma and signs of airway inflammation.

### Patient preferences and commitment as an essential element of therapy effectiveness supporting the concept of BUD-FORM as needed

For physicians working with patients, solving patents’ everyday problems and meeting expectations are essential elements of asthma management. Understanding the factors that influence patient behavior may help to determine the most effective regimen. Therefore, the results of the study on patients’ adherence to therapy are vitally important.

The Respiratory Patients Opinions Survey (RESPONSE) [54], performed in Europe, showed that the majority of patients prefer to use fewer asthma drugs and to have just one inhaler. Further studies of patient behavior revealed that over half of patients tend to rely on reliever medication [55] and thus underuse ICS [38]. Studies show that fewer than 50% of patients adhere to the prescribed schema [56, 57]. Regardless of the fact that people forget to take drugs regularly, patients are also concerned about the side effects of long-term therapy and dependence [58] and therefore decrease the doses themselves or even stop taking drugs when they feel better. A fixed combination of ICS with RABA as a reliever therapy eliminates the risk of using RABA relievers alone and thus increases safety.

Patients with chronic conditions tend to have a strong influence on the treatment process, regardless of the efficacy of the therapy. Therefore, asthma management must be a compromise between patient and physician preferences. The goal for physicians is complete asthma control, while from a patient’s perspective, limiting the influence of asthma and its therapy on real life is most important. The fixed ICS and formoterol combination has been shown to improve both the safety and the effectiveness of asthma therapy as well as patients’ health-related quality of life. The usage of one inhaler with a simple and intuitive regimen is the most preferred asthma therapy option by both patients and physicians. It also improves anti-inflammatory therapy in mild asthmatics, since patients’ perception of ICS effectiveness is low and often results in the discontinuation of these drugs. Thus, when using a fixed dose combination product, with every rescue inhalation, patients also receive anti-inflammatory treatment.

In 2008, the present authors emphasized that the fixed combination of BUD-FORM as a reliever therapy eliminated the risk of SABA use as monotherapy, thus increasing patient safety. We also stressed that considering a patient’s behavior and preferences is one of the ways to improve a treatment’s effectiveness.

**Fig. 5:**
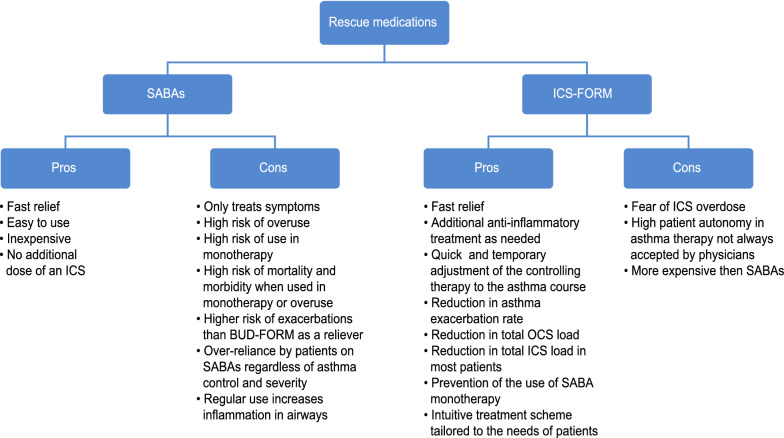
Pro and con for using SABA and ICS-FORM as relivers. *SABA* short acting beta2 agonist, *ICS* inhaled corticosteroids, *FORM* formoterol

This innovative proposal was then criticized by reviewers, mainly due to the lack of registration of the drug for this indication, although the benefits from using BUD-FORM as needed form the 1st step of therapy clearly outweighed the doubts (Fig. 5.)

## New GINA recommendation for mild asthmatics

At the time the 2019 GINA Report was published, neither BUD-FORM nor BDP-FORM had been registered as on-demand relievers when not used in maintenance treatment; nonetheless, the GINA experts issued this recommendation. For safety reasons, GINA no longer recommends using SABAs as monotherapy.

GINA experts recommend low-dose ICS-FORM as needed (off label) as the preferred treatment option during the first step of treatment for patients who suffer from asthma symptoms less than twice per month and who are not at risk of exacerbation. An alternative option is the use of low-dose ICS whenever SABA is taken. Maintenance use of low-dose ICS was recommended in 2014 for patients with risk factors, but this therapy is no longer recommended due to the low rate of compliance in these patients and the risk of exposing them to SABA-only treatment.

In addition to the other preferred options, low-dose ICS-FORM as needed and low-dose ICS and SABA as needed were added by experts to the list of potential options for the second step of treatment. The alternative options include low-dose ICS whenever SABA is taken as well as a leukotriene receptor antagonist.

What changes have caused these recommendations to appear?

## The proof of concept studies for the use of ICS-FORM as a reliever in first and second level therapy

Over the last 10 years, several important clinical trials have been published.

In 2007, Papi et al. [59] published the results of the BEST study. This study showed that in patients with mild asthma, BDP-SALB (Salbutamol) 250/100 μg in a single inhaler, administered as needed, was as effective as the regular use of inhaled BDP (250 micrograms, twice daily) and more effective than as needed SALB in improving morning PEF and in the prevention of exacerbations.

Martinez et al. [60] demonstrated in the TREXA study that in children with mild persistent asthma, the most effective therapy to prevent exacerbations is regular, low-dose ICS (BDP 40 μg 2 inhalations/day); however, ICS as a rescue medication along with SABA (BDP 80 μg for each dose of SALB PRN) could be an effective strategy for the prevention of exacerbations in children with well-controlled mild asthma and is more effective than SABA monotherapy. This new regimen allows children to avoid daily inhaled corticosteroid treatments and their related side-effects, such as growth impairment.

Lazarinis et al. [61] demonstrated that combined BUD-FORM (200/6 μg#) on demand improves asthma control by reducing exercise-induced bronchoconstriction in the same order of magnitude as regular budesonide (400 μg* once daily) treatment and terbutaline on demand, despite a substantially lower total steroid dose. Both these treatments were superior to terbutaline alone on demand, which did not alter the bronchial response to exercise.

A post hoc analysis of the START study results carried out by Reddel et al. [62] showed that the use of low-dose ICS (BUD 200 μg*/day in children or 400 μg*/day in adults), even in patients with sporadic symptoms (zero to one time per week), reduced the risk of exacerbating the disease by half compared to patients using only SABA.

Two studies published in 2018, SYGMA 1 and 2, seem to have been decisive for the change in GINA recommendations. O’Byrne et al. [63] (SYGMA 1) demonstrated that in patients with mild asthma, a fixed combination of BUD-FORM (200/6 μg*) used as needed reduced the frequency of exacerbations by 64% compared to SABA monotherapy. Moreover, the studies by O’Byrne (SYGMA 1) and Bateman (SYGMA 2) [64] both showed that a BUD-FORM treatment regimen (200/6 μg* PRN) protected mild asthmatics against exacerbations equally as effectively as low-dose ICS (BUD 200 μg* BID and SABA as a rescue).

## The reasons for changes in the GINA guidelines

The results of these studies coincided with the publication of alarming epidemiological data from England and Wales, which showed the growing trend of deaths from asthma. In the last decade, the number of deaths due to asthma has increased there by 25%, reaching the highest number per year in this century [65].

Since effective, safe medications are available and the recommendations for how to effectively treat asthma are commonly known, what is causing this increase?

One of the problems seems to be the overuse of SABA in monotherapy by patients with mild asthma. Therefore, for safety reasons, GINA no longer recommends treatment using only SABA monotherapy in any step.

The introduction of symptom-driven treatment that combines RABA and ICS in the first step of therapy unifies the message (to providers and patients) of the principles of treating this disease, which had up to now been contradictory. From the beginning, it has been commonly known that asthma is an inflammatory disease, but anti-inflammatory drugs were recommended only in the second step of therapy. Another problem was the ambiguous message given to patients: on one hand, the recommendations assumed freedom in the use of drugs by patients during the first step of therapy, but on the other hand, these recommendations were much stricter and more inflexible during the second through fifth steps of therapy.

Experts also emphasize that the recommended changes are meant to counteract the trend of patients who rely solely on rescue medications, which results in the discontinuation of ICS therapy because it is perceived as less effective in treating patients’ symptoms while also carrying risks of side effects. Patients using SABA as monotherapy have unconsciously increased their risk of severe exacerbations. Therefore, the need to track patterns of patient behavior is a necessity to improve the adherence and safety of treatment (Fig. 5).

## BUD-FORM as needed for everyone or for patients with specific asthma phenotypes/endotypes

RABAs are considered the most effective drugs that reverse bronchospasm, and their choice does not require the determination of asthma phenotypes or endotypes. There are studies mention above which show that formoterol is as effective and fast in bronchodilatation as salbutamol [49] and has good or even a better safety profile [48]. The adding of BUD to FORM does not change the bronchodilatory response [50] but guarantees taking of ICS by patients who used relievers. The combination of a low dose of BUD-FORM as a rescue medication is preferred by GINA experts for treatment at the 1st and 2nd steps of treatment and for patients who use the same drug in maintenance therapy from step 3. Another combination—low dose of BDP-FORM can be also used as reliever. SMART/MART therapy is especially recommended for patients with asthma with the phenotype of frequent exacerbations, but it can be used in any patient who requires ICS-LABA [37].

However, neither BUD/BDP-FORM combinations are the only recommended ICS-LABA by GINA experts, nor is BUD-FORM at the 1st and 2nd steps the only recommended therapeutic option as described previously. If other than ICS-FORM combined ICS-LABA is preferred, SABA must be used as reliever. At 1st and 2nd step SABA may be used but always with concurrent use ICS in one or separate inhalers [37].

Leaving the classic regimens for asthma therapy is justified by the availability of medical preparations and their costs, by the patients’ habituation to the traditional treatment regimen and the choice of the suitable drug delivery system, by the lack of SMART/MART effectiveness in certain patients, and finally by the possible but rare intolerance to formoterol.

From the above mentioned reasons, the biggest barrier in popularizing BUD-FORM as a rescue medicine seems to be the habit of patients and physicians holding to traditional regimens of asthma therapy. And so, despite the availability of BUD-FORM in Poland and its reimbursement by the National Health Fund, one-third of patients using low dose BUD-FORM still use SABA as a rescue [66].

## Unmet needs in the GINA guidelines

### This chapter presents the personal views of the authors regarding the needs for the development of asthma guidelines

Current changes to the GINA guidelines in the treatment of patients with mild asthma are long-awaited changes with very strong evidence of efficacy, safety and patient acceptance [37].

However, the new guidelines lack practical advice for doctors and patients to seamlessly switch from the symptom-driven therapy during the first and second steps of treatment to SMART therapy during the third through fifth steps of treatment; furthermore, they do not clearly state when to increase the maintenance dose of ICS during SMART treatment. Perhaps by default, symptoms of uncontrolled asthma, as defined by GINA experts, should be such a signal.

As a reminder, the symptoms of uncontrolled asthma are daytime asthma symptoms appearing more frequently than twice a week, any nighttime waking due to asthma, the need for reliever use due to asthma symptoms more than twice per week, and any limitation to activity due to asthma.

This approach to therapy was previously proposed by the authors in their review in 2008 [46].

Lack of clear indications for asthma treatment intensification may lead to uncertainty and limit the number of providers applying this treatment algorithm on a regular basis.

The second problem is the maintenance of SABA monotherapy in children during step one of treatment, despite studies showing the beneficial effects of symptom-driven therapy with ICS in this age group and the popularity of this pattern with pediatricians. Declarations on the shift from asthma being sheer bronchoconstriction to asthma as an inflammatory disease do not seem to apply to children.

Finally, the authors believe that the symptom-driven approach in asthma therapy would be easier to implement if the new approach was clearly separated from previously recommended treatments by modifying the main GINA asthma management graph. This would provide greater clarity regarding the new methods and would emphasize the flexibility of ICS doses, which means a fluid rather than a rigid stepwise transition to the next level of therapy.

**Fig. 6:**
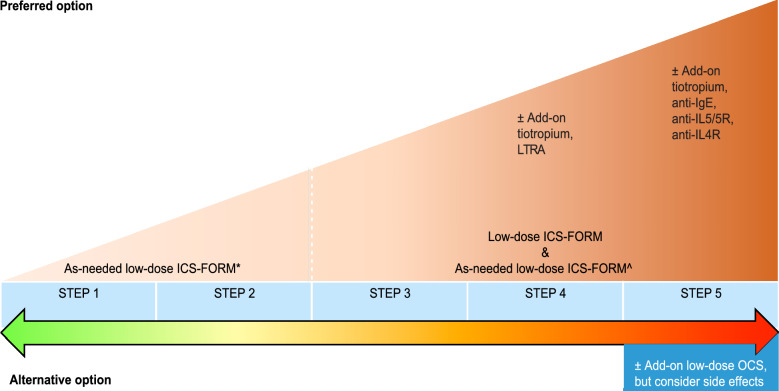
Symptom-driven approach. *ICS* inhaled corticosteroids, *FORM* formoterol

**Fig. 7:**
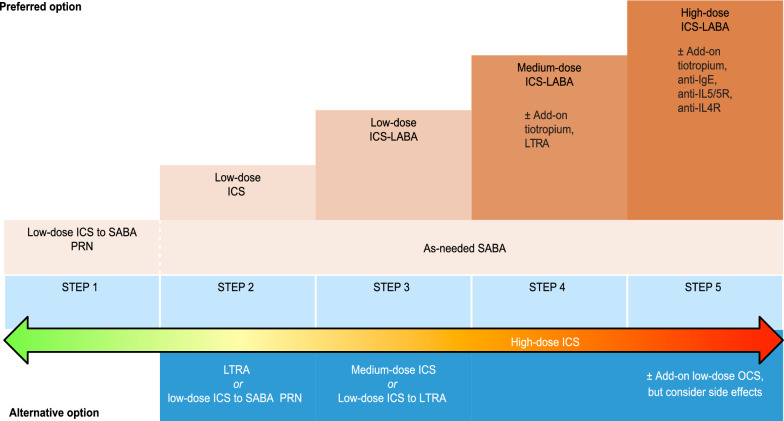
Conventional approach. *ICS* inhaled corticosteroids, *LABA* long acting beta2 agonist, *SABA* short acting beta2 agonis

The authors’ proposed graphs can be found below: Fig. 6 (the new approach) and Fig. 7 (the conventional approach).

And the last problem to think about for future is whether biological therapies should be really at the end of the therapeutic ladder. In patients with severe asthma, the pathomorphological and pathophysiological changes may be irreversible. Earlier introduction of biological therapies could prevent the development of severe asthma as well as complications after OCS and high doses of ICS. It may also lead to a full remission of the disease as it was observed in anti-IgE therapy in children [67] and perhaps may be available in coming soon anti-TSLP or anti-IL-33 therapy. Due to the high costs of these therapies and the problematic route of administration, they may not gain acceptance in mild asthma, but if every day good asthma control is interrupted by severe life-threatening attacks, it may be worth considering anti-IgE in such asthmatics with an allergic background.

## Summary

Current changes in the asthma recommendations have been long-awaited and are focused on reducing the risk of morbidity and mortality, which have been repeatedly emphasized by experts. One might wonder why these changes were introduced so late, since it has been known for many years that the use of SABA monotherapy is associated with a serious risk of exacerbations and death. A fixed combination of ICS and RABA has been available for many years, and it has been known that this combination increases the safety of patients, although there is also an option to simultaneously use ICS and RABA from separate inhalers. One of the explanations for the delay in changing the recommendations is that evidence from clinical trials is needed in order to make positive changes, and the results from clinical trials require years of analysis. Therefore, we are witnessing an evolution rather than a revolution in changing treatment patterns. Undoubtedly, an important lesson from the implementation of these asthma guidelines is that the careful observation of patients’ behavior can contribute to significant progress in the treatment of chronic diseases. The effectiveness of treatment not only depends on the efficacy of the drug itself and the systems for its administration, but it also depends on the patient’s acceptance and compliance. Treatment does not depend solely on the best medicine being written on the prescription, but the one bought by the patient and used as recommended. The use of SABA monotherapy in the treatment of chronic asthma is no doubt falling out of favor, but in order to completely eliminate this treatment, the popularity of using combined preparations of ICS-RABA must be increased.

For those who are still unconvinced, new changes in the GINA guidelines, which are symptom-driven during the first and second steps of therapy together with SMART treatment during the third through fifth steps, create the most personalized asthma therapy we know, regardless of asthma phenotype and endotype, where the ICS dose is best-suited to asthma activity. It allows treatment with the lowest dose of ICS, which ensures a low risk of side effects (which is extremely important for long-term treatment), while maintaining effectiveness to ensure unhampered life activities and preventing severe exacerbations. In addition, that is why, until the discovery of a drug that will cure asthma (which will be revolutionary), the symptom-driven approach in asthma management, according to the authors, is the best therapeutic option and the most personalized option currently available for the majority of patients.

## Conclusions

1. Many epidemiological, clinical and experimental data have shown that SABA monotherapy and/or overuse are associated with a serious risk of exacerbations and death.
2. Changes proposed in the 2019 GINA report (withdrawal of the recommendation for SABA in monotherapy and the introduction of symptom-driven ICS-RABA therapy on 1st and 2nd step) are the most fundamental change to asthma therapy in the last 30 years.
3. A fixed combination of ICS and RABA prevents intentional and unintentional using SABA in monotherapy by patients and therefore increases their safety.
4. Therapy tailored to the patient’s behavior improves the effectiveness of asthma treatment.
5. There are still several gaps in recommendation, the lack of the practical tips when to switch from the symptom-driven to SMART therapy and no symptom-driven approach in younger children which must be resolved in the nearest future.

## Data Availability

All papers discussed in the review are accessible through medical databases and are listed in the references.

## Abbreviations

#: Metered dose
^: Delivered dose
*: Off-label, data for BUD-Form; BDP-FORM or BUD-Form
BAL: Bronchoalveolar lavage
FeNO: Exhaled nitric oxide
GINA: Global Initiative for Asthma
ICS: Inhaled corticosteroids
LABA: Long acting beta2 agonist
MBP: Major basic protein
PRN: As needed
RABA: Rapid acting beta2 agonist
SABA: Short acting beta2 agonist
SMART: Single inhaler maintenance and rescue therapy
